# Exposure to animals and increased risk of marginal zone B-cell lymphomas of the ocular adnexae

**DOI:** 10.1038/bjc.2012.2

**Published:** 2012-01-24

**Authors:** R Dolcetti, D Serraino, G Dognini, S Govi, R Crocchiolo, P Ghia, E Pasini, M Ponzoni, R Talamini, P De Paoli, C Doglioni, A J M Ferreri

**Affiliations:** 1 Centro di Riferimento Oncologico - IRCCS, National Cancer Institute, 33081, Aviano, Italy; 2 Unit of Lymphoid Malignancies, Department of Onco-Hematology, San Raffaele Scientific Institute, 20132, Milan, Italy

**Keywords:** non-Hodgkin's lymphomas, ocular adnexal marginal zone B-cell lymphoma epidemiology, animal exposure, *Chlamydophila psittaci*

## Abstract

**Background:**

Ocular adnexal marginal zone B-cell lymphoma (OAMZL) has been associated with *Chlamydophila psittaci*, an infection that may be transmitted by carrier animals. However, it is still unclear whether exposure to animals affects the risk of OAMZL in comparison with other lymphoma histotypes. We therefore investigated the role of professional and/or domestic exposures to animals in the occurrence of OAMZL, as compared with other types of lymphoma.

**Methods:**

A hospital-based case–control study was carried out on 43 consecutive OAMZL patients (cases) and 87 consecutive patients with nodal non-Hodgkin's lymphomas (NHLs; controls). Multiple logistic regression (MLR) odds ratios (ORs), and 95% confidence intervals (CIs) were used to estimate the association between exposures to animals and OAMZL risk.

**Results:**

A higher proportion of cases reported a lifetime exposure to household animals (79.1% *vs* 64.4% among controls), with a non-statistical significant MLR-OR of 2.18 (95% CI: 0.85–5.62). The OAMZL cases more frequently reported a history of occupation in breeding and/or slaughtering than controls (34.9% *vs* 6.9%), with an overall increased risk of 7.69 (95%CI: 2.65–22.34).

**Conclusion:**

These results indicate that, compared with nodal NHLs, the risk of OAMZL is markedly increased by contact with animals, particularly by occupational exposures.

Non-Hodgkin's lymphomas (NHLs) constitute a heterogeneous group of malignancies whose aetiology remains elusive (Blinder and Fisher, 2008). The incidence of NHLs has risen steadily in many countries during the last decades, although this cannot be entirely attributable to improved diagnosis or classification (Adamson *et al*, 2007; Bosetti *et al*, 2008). Underlying autoimmunity and exposure to environmental, occupational, or infectious agents have been suggested to increase the risk of NHLs (Guidoboni *et al*, 2006; Alexander *et al*, 2007; Blinder and Fisher, 2008; Hjalgrim and Engels, 2008; Marcucci and Mele, 2011). Several animals may be carriers of lymphomagenic infectious agents, and indeed elevated risks have been variably associated with contacts to livestock (Amadori *et al*, 1995; Svec *et al*, 2005; Tranah *et al*, 2008), or with occupational exposure to meat (Fritschi *et al*, 2002; McLean and Pearce, 2004). However, as available evidence is inconclusive (Moore *et al*, 2007), as a likely consequence of NHL heterogeneity, subtype-specific aetiological research is required.

Ocular adnexal lymphomas represent relatively rare lymphoproliferative disorders whose incidence is rising (Moslehi *et al*, 2006). Extranodal marginal zone B-cell lymphoma (ocular adnexal marginal zone B-cell lymphoma (OAMZL)) is the commonest entity, and it has been strongly associated with *Chlamydophila psittaci* (*Cp*) infection (Ferreri *et al*, 2004, 2009). This association has been demonstrated with several detection methods, including different PCR protocols, immunohistochemistry, immunofluorescence, and electronic microscopy (Ponzoni *et al*, 2008). Importantly, viable *Cp* has been *in vitro* isolated from OAMZL patients (Ferreri *et al*, 2008). *Chlamydophila psittaci* is the aetiological agent of psittacosis, a human zoonotic disease caused by exposure to infected animals, mostly birds but also domestic mammals and pets (Beeckman and Vanrompay, 2009; Sprague *et al*, 2009). A previous investigation has suggested that contact with household animals is more common among OAMZL patients than in healthy individuals (Ferreri *et al*, 2008). It remains to be clarified, however, whether exposure to animals affects the risk of OAMZL in comparison with patients affected by other types of NHLs. We therefore investigated the role of professional and/or domestic exposures to animals in the occurrence of OAMZL, as compared with the occurrence of other types of lymphoma.

## Patients and Methods

A hospital-based, case–control study was conducted at the San Raffaele Scientific Institute, Milan, Northern Italy, after approval by the local IRB, between June 2004 and December 2009. Data were from 43 consecutive OAMZL patients (the cases; median age: 60.0 years) and 87 consecutive control patients with nodal NHLs (median age: 65.0 years; Table 1). A standardised and validated questionnaire was used to investigate the lifetime history of occupational and domestic exposure to cats, dogs, birds, and other pets (all vertebrates included). All cases and controls were HIV-negative, while 31/43 OAMZL cases were *Cp*-associated, as demonstrated by the presence of *Cp* DNA in tumour tissues (Ferreri *et al*, 2004). Multiple logistic regression (MLR), odds ratios (ORs), and 95% confidence intervals (CIs) were used to estimate the association between the above-mentioned exposures and the occurrence of OAMZL (Breslow and Day, 1980).

## Results

Overall, lifetime exposure to domestic animals was more frequently reported by cases (79.1%) than controls (64.4%), with a non-statistical significant 2.2-fold increased risk of OAMZL (Table 2). The risk of OAMZL was significantly associated with domestic exposure to dogs, with a 3-fold increase (95% CI:1.00−9.05). Conversely, increased, but non-statistically significant, ORs emerged with regard to exposure to cats and birds.

As compared with 6.9% of controls, 34.9% of cases reported a lifetime occupational exposure to animals (MLR-OR:7.69; 95% CI: 2.65–22.34). In particular, 27.9% of cases were involved in slaughtering with a MLR-OR of 16.65 (lower 95% CI: 3.47; Table 2). These results were confirmed when the analysis was restricted to the 31 *Cp*-associated OAMZL (data not shown in tables). Lifetime exposure to all types of domestic animals was associated with a 2.44-fold higher risk of OAMZL (95% CI:0.78–7.57), with non significantly elevated risks for exposure to cats (MLR-OR=3.29) and/or dogs (MLR-OR=3.15). Significantly elevated MLR-OR were noted among *Cp*-positive cases for occupational exposure, with a 9-fold increase for all types of exposure (MLR-OR=9.22, 95% CI: 2.92–29) and a 21-fold higher risk associated with slaughtering (95% CI: 4.21–107; data not shown in tables).

The analysis was also stratified according to the histological diagnosis of controls, by separately comparing the 43 OAMZL cases with the 49 controls diagnosed with a diffuse large B-cell lymphoma (DLBCL), or with the 32 controls diagnosed with a follicular lymphoma (FL; Table 3). The comparison with both control groups confirmed the increased risks in OAMZL patients associated with overall occupational exposure to animals (MLR-OR=8.61 and MLR-OR=9.26 for DLBCL and FL, respectively) and with slaughtering in particular (MLR-OR=18.18 and MLR-OR=13.89 for DLBCL and FL, respectively). No statistically significant elevated risks were found for breeding or exposures to domestic animals (Table 3).

## Discussion

To our knowledge, this is the first epidemiological investigation suggesting that contact with animals is associated with increased risk of OAMZL, even in comparison with other NHL entities. This association seems to be particularly strong for occupational exposure to animals or their products. Previous studies reported a globally increased risk of NHL among individuals exposed to livestock, mainly cattle and pigs (Amadori *et al*, 1995; Svec *et al*, 2005; Tranah *et al*, 2008), or working with meat (Fritschi *et al*, 2002; McLean and Pearce, 2004), although these associations were not confirmed (Holly *et al*, 1997; Persson and Fredrikson, 1999; Dryver *et al*, 2004; Moore *et al*, 2007). These inconsistencies may be due to the fact that NHLs were considered as a single entity or that only the major NHL histotypes were analysed. In this study, the analysis of a relatively large series of NHL of a distinct histotype and arising from the same extra-nodal district allowed the identification of a previously unrecognised association of OAMZL with occupational exposure to animals, highlighting a markedly increased risk. This was particularly evident for slaughterhouse workers, both overall and when cases were separately compared with DLBCL controls or FL controls. This observation reinforced the notion that working in the meat industry entails several potentially hazardous exposures, potentially including infectious agents. Available evidence, however, does not support a pathogenic role for zoonotic viruses, such as retroviruses and herpes viruses, specific to beef cattle and chicken (Bender *et al*, 1988; Lee *et al*, 2005). The associations observed in our study were even stronger when the analysis was restricted to *Cp*-positive case, thus pointing to the transmission of *Cp* infection as a likely relevant risk factor.

Our findings also provide novel insights into the still controversial issue of the association between contacts with pets and NHL risk (Holly *et al*, 1997; Persson and Fredrikson, 1999; Dryver *et al*, 2004; Tranah *et al*, 2008). We found a borderline significant increase of OAMZL risk associated with exposure to one or more domestic animals, especially to dogs. Our results may be consistent with the peculiar pathogenic mechanisms proposed for OAMZL, which likely involve a chronic stimulation by exogenous antigen(s) (Ferreri *et al*, 2009). In this scenario, contacts with domestic animals may favour the transmission of infectious agents that, under still poorly defined circumstances, may trigger and sustain B-cell lymphoproliferation. The further increased risk of OAMZL detected when only *Cp*-associated cases were analysed is consistent with this hypothesis. Intriguingly, we observed an increased risk for *Cp*-associated OAMZL for individuals exposed to dogs, even if these animals are presently not considered the main vehicle of *Cp* transmission to humans. Indeed, *Cp* infection may well be detected in dogs (Fukushi *et al*, 1985; Sprague *et al*, 2009), even in public areas where contamination by potentially zoonotic pathogens seems unlikely (Tarsitano *et al*, 2010). This picture is similar to what is emerging for gastric lymphomagenesis sustained by Helicobacter strains different from *Helicobacter pylori*, which originate from contacts with domestic animals (Haesebrouck *et al*, 2009).

Possible limitations of this study include the relatively undersized investigated sample and the fact that the recruitment of cases and controls was hospital-based. Considering the relative rarity of OAMZL, we were able to collect a series of 43 cases large enough to obtain results of statistical significance with substantial increased risks (i.e., 3-fold or higher after adjustment for potential confounders). It should be admitted, however, that the relative small number of cases limited the analysis stratified by histological types. Although cases and controls were carefully matched for age and sex, a slightly higher fraction of females was included in the case group. This was due to the higher prevalence of OAMZL among women (Ferreri *et al*, 2009).

In conclusion, this case–control study indicates that domestic or occupational contacts with animals increase the risk to develop OAMZL. These findings further stimulate new investigations aimed at identifying the aetiologic agents related to this exposure and involved in OAMZL pathogenesis.

## Figures and Tables

**Table 1:** Description of the study population according to socio-demographic characteristics and tumour histology (Italy, 2004–2009)

	**Cases (*n*=43)**	**Controls (*n*=87)**	
	**No. (%)**	**No. (%)**	***P*-value**
*Sex*			
Female	29 (67.4)	44 (50.6)	
Male	14 (32.6)	43 (49.4)	0.10
			
*Age*			
⩽54	16 (37.2)	23 (26.4)	
55–69	15 (34.9)	32 (36.8)	
⩾70	12 (27.9)	32 (36.8)	0.40
			
*Years of education* a			
⩽5	9 (22)	23 (28.4)	
6–13	26 (63.4)	51 (63.0)	
⩾14	6 (14.6)	7 (8.6)	0.51
			
*Lymphoma histology* b			
Extranodal marginal zone lymphoma	43 (100)	—	
Nodal marginal zone lymphoma	—	4 (4.6)	
Diffuse large B-cell lymphoma	—	49 (56.3)	
Follicular lymphoma	—	32 (36.8)	
Small lymphocytic lymphoma	—	2 (2.3)	

a The sum does not add up to the total because of missing values.

b All diagnoses were confirmed by central pathology review.

**Table 2:** Association between exposure to animals and diagnosis of ocular adnexal lymphoma (Italy, 2004–2009)

	**Cases no. (%)**	**Controls no. (%)**	**MLR-OR (95% CI)** a
*History of exposure to domestic animals*			
No	9 (20.9)	31 (35.6)	1
Yes, all	34 (79.1)	56 (64.4)	2.18 (0.85–5.62)
Cats	20	34	2.29 (0.84–6.28)
Dogs	19	25	3.00 (1.00–9.05)
Birds	18	26	2.00 (0.67–5.97)
			
*History of occupational exposure to animals*			
No	28 (65.1)	81 (93.1)	1
Yes, all	15 (34.9)	6 (6.9)	7.69 (2.65–22.34)
Slaughter	12	2	16.65 (3.47–80.02)
Breeding	3	4	2.65 (0.53–13.17)

a Multiple logistic regression (MLR) odds ratios (ORs) adjusted for sex, age and, when appropriate, history of occupational exposure to animals (no/yes).

**Table 3:** MLR, ORs, and 95% CIs for developing OAMZL by exposures to animals and histological diagnosis of controls (Italy, 2004–2009)

	**MLR-OR (95% CI)^a^**	
	**DLBCL NHL controls**	**FL NHL controls**
*History of exposure to domestic animals*		
No	1	1
Yes, all	1.47 (0.47–4.54)	1.74 (0.51–5.88)
Cats	1.80 (0.53–6.06)	1.83 (0.47–7.11)
Dogs	1.48 (0.35–6.27)	2.91 (0.03–11.65)
		
*History of occupational exposure to animals*		
No	1	1
Yes, all	8.61 (2.09–36)	9.26 (1.90–45)
Slaughter	18.18 (2.13–142)	13.89 (1.66–111)
Breeding	2.81 (0.37–21)	4.76 (0.45–50)
Birds	2.15 (0.47.9.82)	1.48 (0.37–5.86)

Abbreviations: CI=confidence intervals; DLBCL=diffuse large B cell lymphoma; FL=follicular lymphoma; MLR=multiple logistic regression; ORs=odds ratios; OAMZL=ocular adnexal marginal zone B-cell lymphoma.

a MLR ORs adjusted for sex, age and, when appropriate, history of occupational exposure to animals (no/yes).
